# The diagnostic accuracy of magnetic resonance imaging for anterior cruciate ligament injury in comparison to arthroscopy: a meta-analysis

**DOI:** 10.1038/s41598-017-08133-4

**Published:** 2017-08-08

**Authors:** Kun Li, Jun Du, Li-Xin Huang, Li Ni, Tao Liu, Hui-Lin Yang

**Affiliations:** 1 Department of Orthopedic Surgery, First Affiliated Hospital of Soochow University, Suzhou, 215000 China; 2 Department of Orthopedic Magnetic Resonance Chamber, First Affiliated Hospital of Soochow University, Suzhou, 215000 China

## Abstract

We performed this meta-analysis to examine the diagnostic accuracy of MRI for the diagnosis of anterior cruciate ligament (ACL) injury in comparison to arthroscopy. We also compared the diagnostic accuracy of MRI with magnetic field intensities (MFI) greater than or equal to 1.5T with those below 1.5T, in addition to different MRI sequences. Studies relevant to the diagnosis of ACL injury by MRI and arthroscopy were analyzed. Computer and manual retrieval were carried out on studies published between January 1, 2006 and May 31, 2016. Twenty-one papers were included. Neither threshold nor non-threshold effects were present (*p* = 0.40, *p* = 0.06). The pooled sensitivity (SE), specificity (SP), positive likelihood ratio (LR+), negative likelihood ratio (LR−) and diagnostic odds ratio (DOR) with 95% confidence interval (CI) were 87% (84–90%), 90% (88–92%), 6.78 (4.87–9.44), 0.16 (0.13–0.20) and 44.70 (32.34–61.79), respectively. The area under the curve (AUC) was 0.93. The risk of publication bias was negligible (*p = *0.75). In conclusion, examination by MRI is able to provide appreciable diagnostic performance. However, the principle, which states that the higher the MFI, the better the diagnostic accuracy, could not be verified. Additionally, conventional sequences (CSs) associated with proton density-weighted imaging (PDWI) are only slightly better than CSs alone, but not statistically different.

## Introduction

The anterior cruciate ligament (ACL) extends from the posterior surface of the medial femoral condyle and attaches to the intercondylar process of the tibia. Its average length is 31–38 mm and its average intersecting surface area is 36 mm^2^ in females and 44 mm^2^ in males^1^. It can be divided into anteromedial and posterolateral bundles. The main function of the ACL is to limit the forward slip of the tibia on the femur. The anteromedial bundle of the ACL can prevent excessive external rotation of the leg, whereas the posterolateral bundle prevents excessive internal rotation. With the posterior cruciate ligament, the ACL limits excessive flexion, and excessive extension in combination with the posterior cruciate ligament, the medial and lateral collateral ligaments, the articular capsule and the oblique popliteal ligament^2^. It also contributes to restriction of lateral slip and rotation with the articular capsule, the medial and laterial collateral ligaments and the posterior cruciate ligament^3^.

Injuries of the ACL are generally sports-related traumas with nearly three quarters being non-contact^4^. They are a major problem worldwide with approximately 200,000 cases per year in the United States alone^5^. Isolated injuries of the ACL are most frequently caused by forced extension stress associated with “kissing contusions” of the anterior portion of the lateral femoral condyle and of the lateral tibial plateau. It is less frequently caused by forced flexion stress associated with avulsion fracture of the tibial eminence. Associated injuries of the ACL and other structures are caused by a variety of events: (i) ACL injury associated with medial collateral ligament and medial meniscus injury is caused by forced flexion-external rotation stress; (ii) damage associated with lateral compartment injury is caused by forced flexion-internal rotation stress; (iii) when associated with lateral and medial compartment injury it is caused by different associations of varus-valgus and rotatory stress; (iv) when associated with posterior cruciate ligament injury it is caused by posterior displacement of the tibia and knee hyperextension^4, 6^.

Arthroscopy allows direct visualization of all intra-articular structures and thus provides a high level of accuracy for both diagnosis and treatment, which makes arthroscopy the gold standard for evaluation of internal disorders and other lesions of the knee^7^. However, arthroscopy constitutes a relatively expensive and invasive examination^8^. Besides, it is less effective for the assessment of extracapsular soft tissues^9^.

Magnetic resonance imaging (MRI) is a non-invasive method with good soft tissue contrast, high spatial resolution, multi-parameter and multi-range imaging for the evaluation of knee lesions^10^. It can clearly display the injury site of the ACL, the extent of the damage, the degree of injury and the damage to the related structures. Although MRI has recently played an increasing role in the evaluation of knee lesions, its diagnostic potential for ACL injury is limited and diagnosis fallible^9^.

Recent studies have compared the diagnostic accuracy of 1.5T MRI with 3.0T MRI^11^, MRI combined with ultrasonography^12^, and MRI combined with physical examination^8, 13^. However, the exact diagnostic accuracy of MRI for ACL injury is unknown, as are the differences in diagnostic accuracy between MRI with magnetic field intensities (MFI) greater than or equal to 1.5T compared with those below 1.5T, or between different MRI sequences, The objectives of this meta-analysis were to (i) systematically examine the diagnostic accuracy of MRI for the diagnosis of ACL injury; (ii) compare the diagnostic accuracy of MFI greater than or equal to 1.5T with MFI below 1.5T; and (iii) compare the diagnostic accuracy of different MRI sequences.

## Results

### Study selection

**Figure 1:**
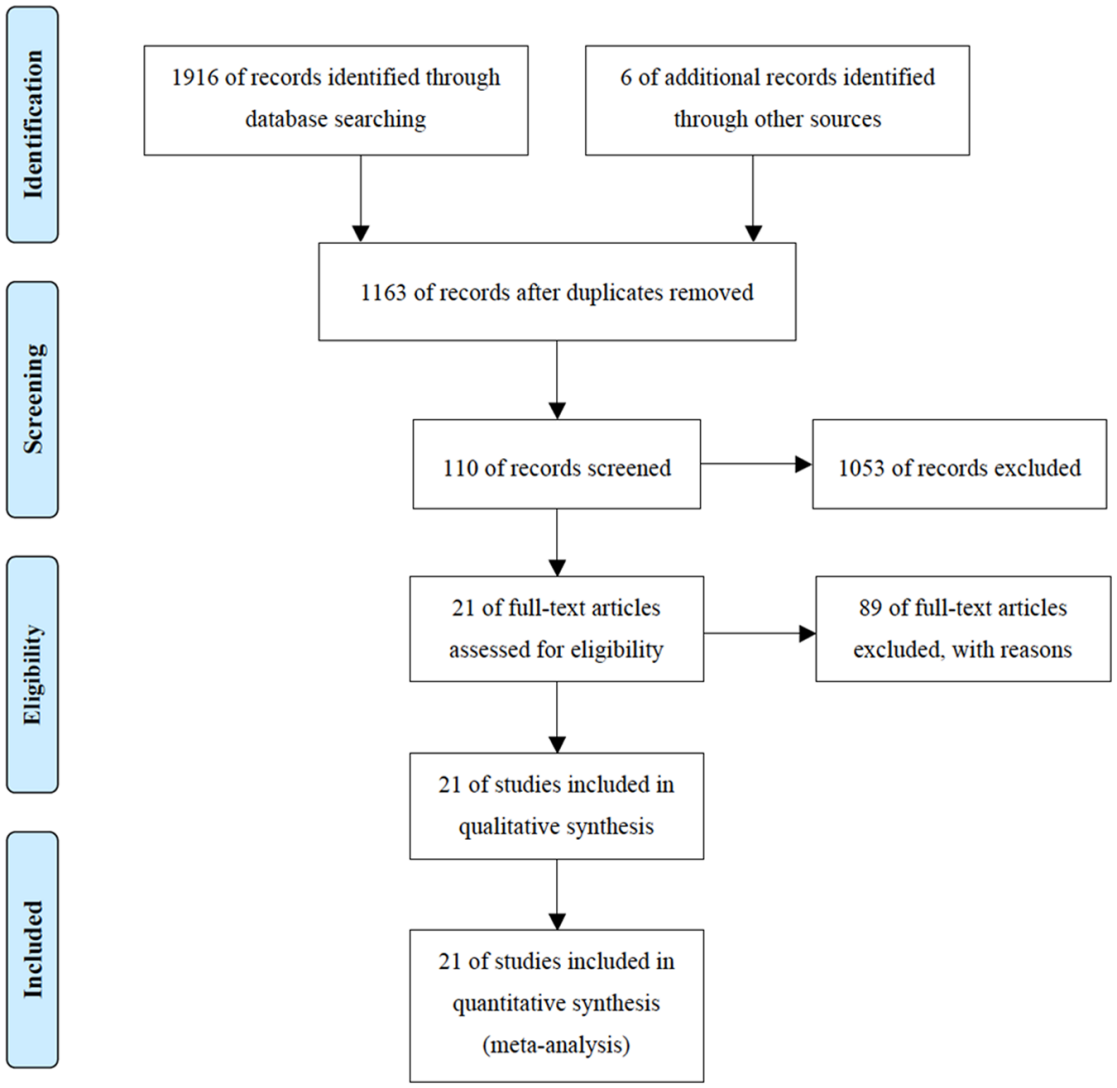
Flow of information through the different phases of the meta-analysis. From: Moher, D., Liberati, A., Tetzlaff, J. & Altman, D. G. Preferred reporting items for systematic reviews and meta-analyses: the PRISMA statement. PLoS medicine 6, e1000097, doi:10.1371/journal.pmed.1000097 (2009).

A total of 1922 articles were initially retrieved for this meta-analysis: 481 from PubMed, 783 from EMBASE, 470 from Ovid, 129 from BIOSIS Previews, 53 from the Cochrane library and 6 articles obtained from manual retrieval of relevant references by sending e-mails to authors. 759 reports were then eliminated out of 1232 duplicated reports as they originated from the same team or the same set of data. According to the inclusion and exclusion criteria for the initial screening, a total of 110 articles were thus selected after reading the title and abstract (71 from PubMed, 24 from EMBASE, 11 from Ovid and 4 from the Cochrane library) and the articles were marked with 1 star in EndNote software. By evaluating the full text, two researchers (K.L. and J.D.) then selected 31 papers that strictly complied with the inclusion and exclusion criteria and marked them with 2 stars in EndNote. Ten studies were excluded after re-assessing the full text during the third screening. Finally, 21 articles^9, 14–33^ were chosen and marked with 3 stars, articles for which true positive (TP), false positive (FP), true negative (TN) and false negative (FN) results could be extracted or accurately calculated through 2 × 2 contingency tables (16 from PubMed and 5 from EMBASE). These articles consisted of 16 prospective studies and 5 retrospective studies, for a total of 1722 cases. The literature search, the screening process and the results are shown in Fig. 1. The basic characteristics of the studies which were included are displayed in Table 1. From a chronological point of view, 38% (8/21) of these studies were published between 2006–2009 inclusive and 62% (13/21) in the period 2012–2016.

**Table 1:** Basic characteristics of the studies included in the meta-analysis.

Included studies	PD	Country	SD	MFI	Study population			Age (year)	Blinding	TP	FP	FN	TN	SE (%)	SP (%)
					NO.	M	F								
S. Thomas *et al*.	2007	UK	RC	1.5T	102	74	27	42	SB	7	5	4	86	63.6	94.5
Mohammed Azfar Siddiqui *et al*.	2013	India	PC	1.5T	51	31	20	41	SB	8	2	1	40	88.9	95.2
Fritz K.W. Schaefer *et al*.	2006	Germany	PC	1.5T	31	16	15	41	DB	16	0	3	12	84.2	100
F. Rayan *et al*.	2009	UK	PC	—	131	—	—	—	DB	22	4	5	100	81.5	96.2
Nilton Orlando Júnior *et al*.	2015	Brazil	PC	—	72	61	11	34	SB	46	5	7	14	86.8	73.7
M. J. Sampson *et al*.	2008	Ireland	PC	3T	61	52	9	30	SB	28	0	0	33	100	100
Amir Mohammad Navali *et al*.	2013	Iran	PC	—	120	108	12	29	DB	71	8	1	40	98.6	83.3
Christopher S. Lee *et al*.	2013	USA	RC	0.2T	379	206	173	48	DB	46	21	8	304	85.2	93.5
Hristijan Kostov *et al*.	2014	Macedonia	PC	1.0T	103	81	22	30	DB	60	5	13	25	82.2	83.3
Gul-e-khanda *et al*.	2008	Pakistan	PC	1.5T	50	32	18	—	DB	13	3	2	32	86.7	91.4
Hayat Ahmad Khan *et al*.	2015	India	PC	—	26	20	6	—	—	16	2	0	8	100	80
I. C. Helmark *et al*.	2007	Denmark	PC	1.5T	50	25	25	28	DB	15	10	2	23	88.2	69.7
Gupta M. K. *et al*.	2014	Nepal	PC	0.35T	40	24	16	30	DB	21	2	2	15	91.3	88.2
Predrag Grubor *et al*.	2013	Bosnia and Herzegovina	PC	—	63	50	13	37	—	21	12	4	26	84	68.4
Julian Dutka *et al*.	2012	Poland	RC	—	113	—	—	37	SB	57	6	14	36	80.3	85.7
Sylvain R. Duc *et al*.	2008	Switzerland	PC	1.5T	60	15	14	41	SB	14	0	6	40	70	100
Peter De Maio *et al*.	2014	Canada	PC	1.5T	50	28	22	41	DB	17	1	0	32	100	97
J. Challen *et al*.	2007	Australia	RC	1.5T	44	25	19	41	SB	10	5	3	26	77	83.9
Francisco Abaeté Chagas-Neto *et al*.	2016	Brazil	PC	1.5T	76	28	10	34	DB	52	4	4	16	92.9	80
Amreen Abdul Bari *et al*.	2014	India	PC	1.5T	71	—	—	—	DB	29	7	4	31	87.9	81.6
T. Ai *et al*.	2012	USA	RC	1.5T	29	17	12	33	DB	10	2	3	14	76.9	87.5

PD: publication date; SD: study design; RC: retrospective cohort; PC: prospective cohort; MFI: magnetic field intensity; No.: number; M: male; F: female; SB: single blind; DB: double blind; TP: true positive; FP: false positive; FN: false negative; TN: true negative; SE: sensitivity; SP: specificity.

### Assessment of risk of bias within studies

**Figure 2:**
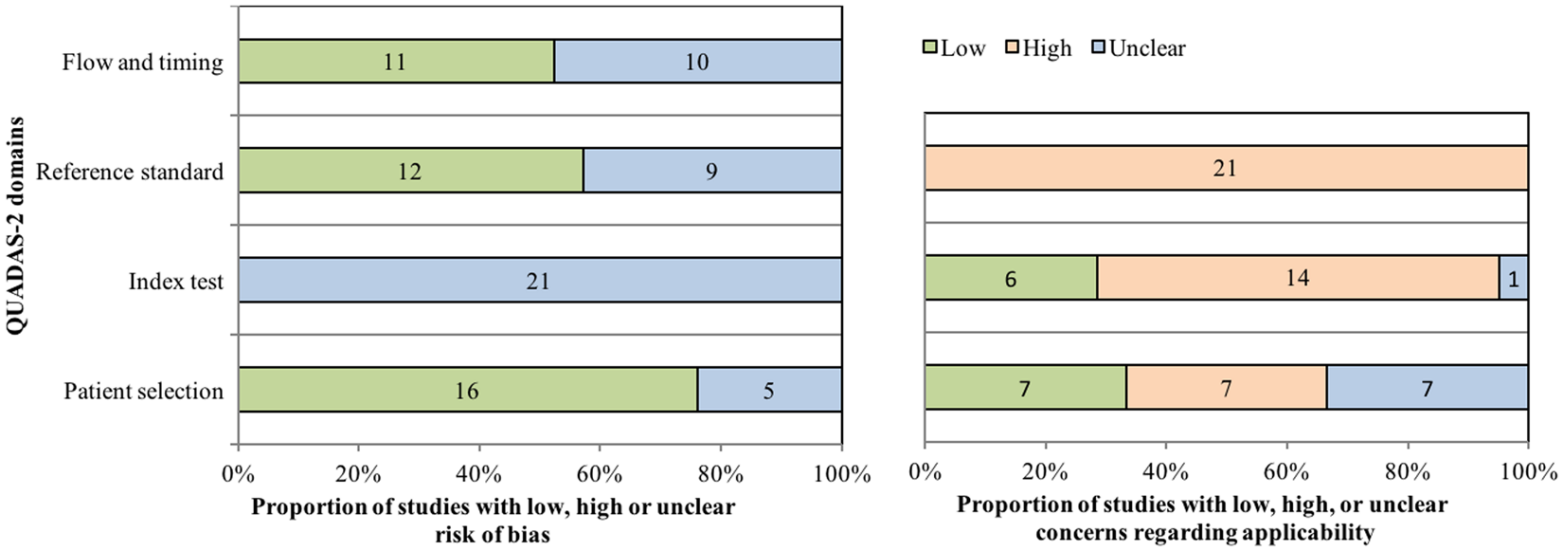
The methodological quality assessment of risk of bias within eligible studies according to QUADAS-2. QUADAS-2: Quality Assessment of Diagnostic Accuracy Studies 2.

The methodological quality assessment of risk of bias within eligible studies is shown in Fig. 2, according to the Quality Assessment of Diagnostic Accuracy Studies 2 (QUADAS-2) tool. Overall, the number of low and unclear risk of bias was 39 and 45, respectively, for the four domains (patient selection, index test, reference standard, and flow and timing). The number of high, unclear and low concerns regarding applicability was 42, 8 and 13, respectively for the three domains (patient selection, index test and reference standard).

### Heterogeneity test of individual studies

**Figure 3:**
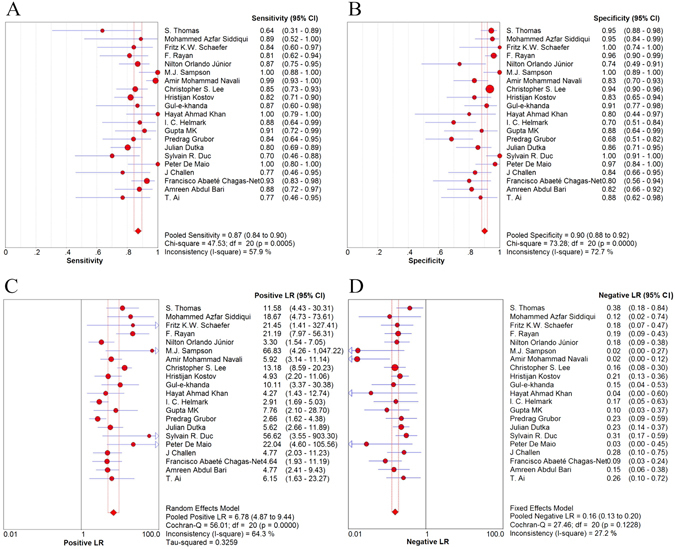
Forest plot of sensitivity, specificity, positive LR and negative LR of MRI for the diagnosis of ACL injury. (**A**) Sensitivity for MRI. (**B**) Specificity for MRI. (**C**) Positive LR for MRI. (**D**) Negative LR for MRI. Corresponding indices, 95% CI and the pooled indices are represented by red circles, horizontal lines and red diamonds, respectively. LR: likelihood ratio; CI: confidence interval.

**Figure 4:**
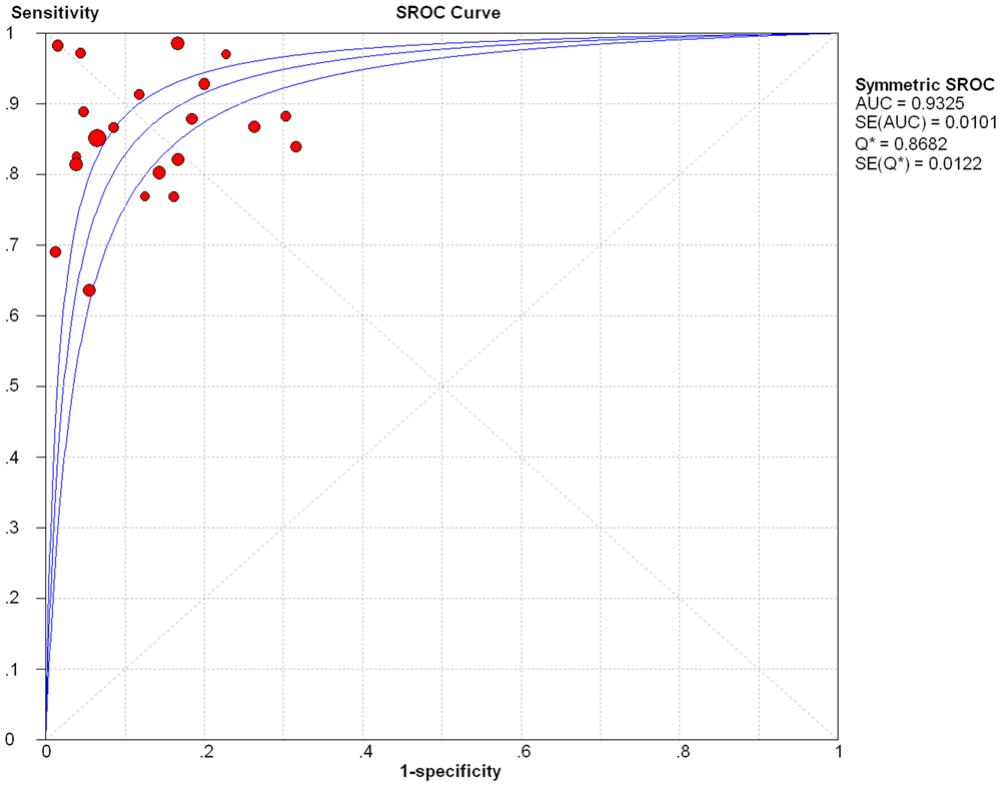
The sROC plane for heterogeneity test of threshold effect of each independent study. The sROC (middle line) with 95% CI (the other two lines) of MRI in diagnosing of ACL injury. sROC: summary receiver operator characteristic; CI: confidence interval; AUC: area under the curve; Q*: Q index value; SE: standard error.

**Figure 5:**
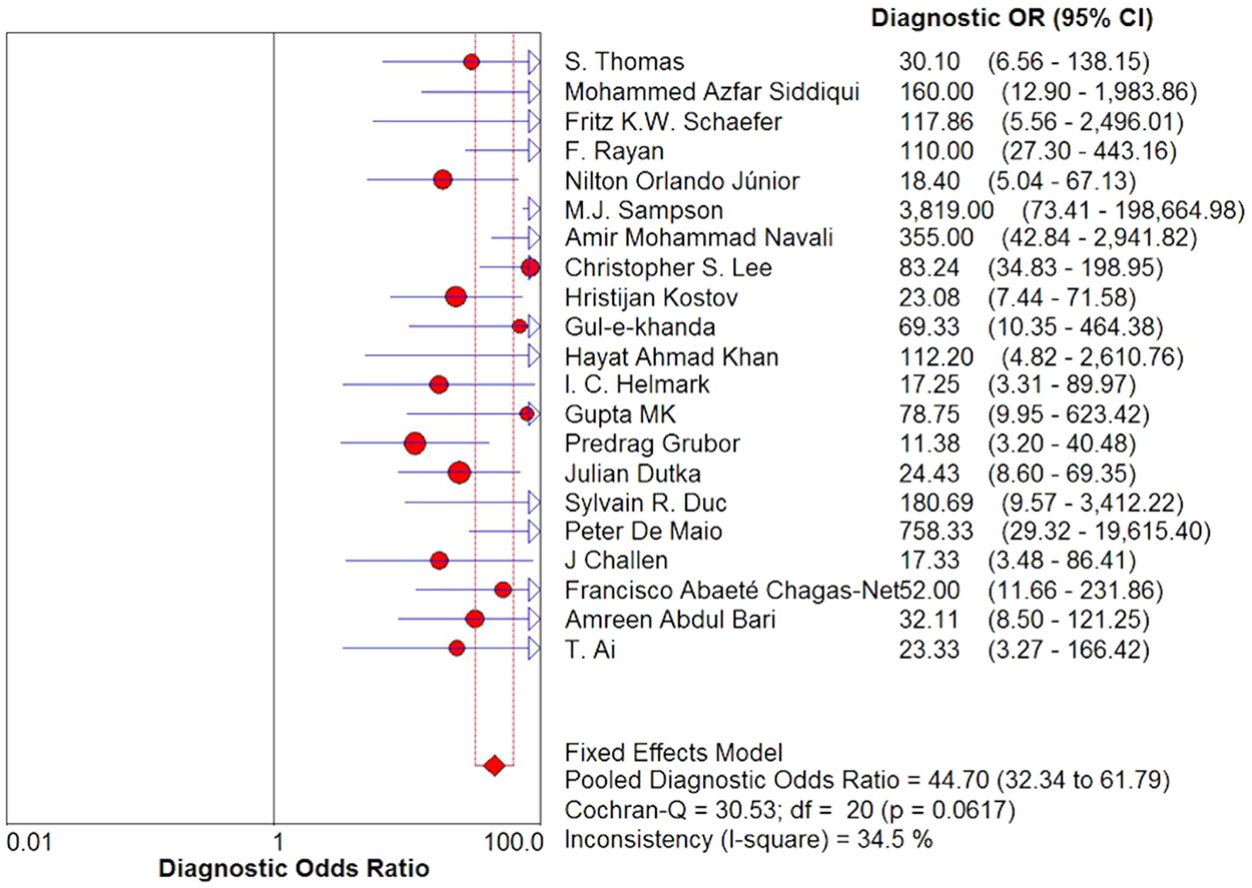
Forest plot of DOR with 95% CI. The DOR, 95% CI and the pooled DOR are represented by red circles, horizontal lines and red diamonds, respectively. DOR: diagnostic odds ratio; CI: confidence interval.

Results of the heterogeneity test for the threshold effect were as follows: the variation tendency of sensitivity (SE) and specificity (SP) or positive likelihood ratio (LR+) and negative likelihood ratio (LR−) were not negatively correlated in forest plots (Fig. 3A–D). The distribution of accuracy estimates of each independent study did not show the “shoulder arm” shape in the summary receiver operator characteristic (sROC) plane (Fig. 4). Results of the Spearman correlation (*p* = 0.40 and *r* = 0.194) between the logit of sensitivity and the logit of 1-specificity indicates that the threshold effect was absent. Regarding the heterogeneity test for the non-threshold effect, results of the Cochran-Q test (*p* = 0.06) indicates that the non-threshold effect was also absent (Fig. 5).

A random effects model was used for pooled SE [*p* < 0.001, inconsistency index (*I* ^2^) = 57.9%], pooled SP (*p* < 0.001, *I* ^2^ = 72.7%) and pooled LR+ (*p* < 0.001, *I* ^2^ = 64.3%) (Fig. 3A–C). A fixed effects model was used for pooled LR− (*p* = 0.12, *I* ^2^ = 27.2%) (Fig. 3D) and pooled diagnostic odds ratio (DOR) (*p* = 0.06, *I* ^2^ = 34.5%) (Fig. 5) respectively. The *I* ^2^ statistics based on Chi square (where Q is the chi-square statistic) was used to quantify the degree of heterogeneity in eligible studies and expresses the percentage of total variation observed across studies caused by heterogeneity rather than by chance. There is no observed heterogeneity when *I* ^2^ = 0, implying that all the variability observed in the effect estimates is due to sampling errors rather than because of heterogeneity amongst trials. Heterogeneity that is low, moderate, or high relates to *I* ^2^ < 25%, 50% < *I* ^2^ < 75%, *I* ^2^ > 75% respectively. Values of *I* ^2^ = 25%, 50% or 75% are defined as 1/4, 1/2 or 3/4 of the variability observed in the effect estimates being attributable to inconsistency among trials.

### Synthesis of results

The pooled SE was 87% [95% confidence interval (CI), 84–90%] and the pooled SP 90% (95% CI, 88–92%), whereas the pooled LR+ was 6.78 (95% CI, 4.87–9.44) and the pooled LR− 0.16 (95% CI, 0.13–0.20) (Fig. 3). The pooled DOR was 44.70 (95% CI, 32.34–61.79) (Fig. 5) and the area under the curve (AUC) was 0.93 (Fig. 4).

### Subgroup analysis

The differences between subgroups were calculated according to the MFI, the year of publication and the type of MRI sequence [conventional sequences (CSs) and CSs with proton density weighted imaging (PDWI)]. The results are listed in Table 2 and include the pooled SE, SP, LR+, LR−, DOR and AUC values.

**Table 2:** Differences between subgroups according to MFI, year of publication and type of MRI sequence.

Category	Studies (n)	Sample size (n)	Threshold effects *p* value	Pooled SE (95% CI)	Pooled SP (95% CI)	Pooled LR+ (95% CI)	Pooled LR− (95% CI)	Pooled DOR (95% CI)	AUC (SE*)
overall	21	1722	0.40	0.87 (0.84, 0.90)	0.90 (0.88, 0.92)	6.78 (4.87, 9.44)	0.16 (0.13, 0.20)	44.70 (32.34, 61.79)	0.93 (0.01)
**MFI**									
≥1.5T	12	675	0.89	0.87 (0.82, 0.91)	0.91 (0.88, 0.93)	7.85 (4.74, 12.99)	0.17 (0.12, 0.23)	50.97 (30.40, 85.46)	0.93 (0.01)
<1.5T	3	522	0.67	0.85 (0.78, 0.90)	0.92 (0.89, 0.95)	8.57 (5.76, 12.74)	0.17 (0.11, 0.26)	50.53 (26.51, 96.30)	0.93 (0.03)
*t* value^*^/*Z* value	—	—	—	0.19^*^	−0.31^*^	−0.29	−0.32^*^	−0.29	—
*p* value	—	—	—	0.85	0.76	0.84	0.75	0.84	—
**Year of publication**									
2006–2009	8	529	0.76	0.83 (0.76, 0.89)	0.93 (0.90, 0.95)	10.83 (4.62, 25.40)	0.20 (0.14, 0.28)	56.63 (30.02, 106.85)	0.92 (0.02)
2012–2016	13	1193	0.82	0.88 (0.85, 0.91)	0.89 (0.86, 0.91)	5.90 (4.08, 8.54)	0.14 (0.11, 0.18)	40.90 (28.02, 59.69)	0.93 (0.01)
*t* value^*^/*Z* value	—	—	—	−1.83^*^	2.07	−1.70	1.67^*^	−0.36	—
*p* value	—	—	—	0.08	0.04	0.09	0.11	0.75	—
**MRI sequences**									
CSs	6	431	0.957	0.87 (0.81, 0.91)	0.88 (0.84, 0.92)	6.31 (3.55, 11.23)	0.17 (0.11, 0.25)	36.62 (19.69, 68.12)	0.92 (0.02)
CSs + PDWI	7	655	0.355	0.87 (0.82, 0.92)	0.92 (0.89, 0.94)	9.54 (6.76, 13.47)	0.15 (0.10, 0.22)	58.07 (33.39, 100.99)	0.94 (0.02)
*t* value^*^/*Z* value	—	—	—	−0.06^*^	−0.10^*^	−0.72	0.13^*^	0.86	—
*p* value	—	—	—	0.95	0.34	0.53	0.90	0.45	—

SE: sensitivity; SP: specificity; LR+: positive likelihood ratios; LR−: negative likelihood ratios; DOR: diagnostic odds ratio; AUC: area under the curve; CI: confidence interval; SE^*^: standard error; MFI: magnetic field intensity; T: Tesla; MRI: magnetic resonance imaging; CSs: conventional sequences; PDWI: proton density weighted imaging; *t*: two independent samples *t*-test; Z: mann-whitney test.

### Publication bias in the literature evaluation

**Figure 6:**
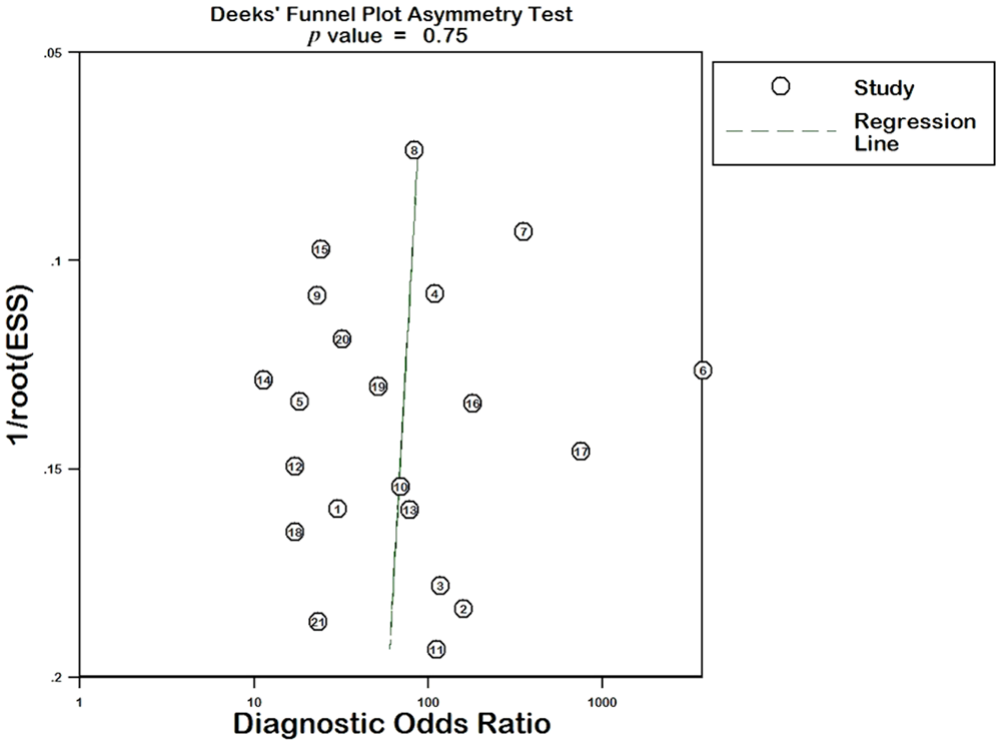
The Deeks’ funnel plot asymmetry test for publication bias in the literature evaluation. Each study is shown as a circle, and the regression line is shown.

The Deeks’ funnel plot asymmetry test for DOR presented basic symmetry (Fig. 6). Nevertheless, results showed no significant risk of publication bias (*p* = 0.75).

## Discussion

Overall, ACL injury is a common clinical form of knee damage. Timely and accurate diagnosis and treatment could prevent the emergence of cartilage degeneration, the progression of bone contusion, the aggravation of traumatic arthritis or the occurrence of knee joint dysfunction^34^.

Magnetic resonance imaging is a noninvasive technique that remains a physician’s first choice for the clinical diagnosis of ACL injury. It has the advantages of good soft tissue contrast, high spatial resolution and allows multi-parameter evaluation of morphological changes in an injured ACL. However, it is likely that overuse of the MRI technique in the diagnosis of ACL injury leads to misdiagnosis (estimated at 47%), especially in a chronic incomplete tear which might be due to the special sensitivity to the hydrogen atom and could be associated with volume effects and synovial hyperplasia^18^. Additionally, different studies have attributed different values for sensitivity and specificity, ranging from 63.6%^14^ to 100%^9, 19, 29^ and from 68.4%^26^ to 100%^16, 19, 28^ respectively, owing to the slightly oblique angle of the ACL crossing the knee joint and to the difficulty of displaying the full ACL in the true sagittal plane via a single MRI scan^22^. Meanwhile, the accuracy of MRI diagnosis depends on the scanning technique and the experience of the musculoskeletal radiologist^30^. Thus, the precise diagnostic accuracy of MRI for ACL injury is unknown. It is necessary, therefore, to carry out high level evidence-based medical research on the accuracy of MRI diagnosis for ACL injury.

Our meta-analysis focused on the diagnostic accuracy of MRI for ACL injury compared with arthroscopy. The pooled SE and SP are 87% (95% CI, 84–90%) and 90% (95% CI, 88–92%) respectively, indicating that the rate of missed diagnosis and misdiagnosis reach 13% and 10%, respectively. Furthermore, a good diagnostic test may have a LR+ superior to 10 and a LR− inferior to 0.1. Our study revealed that the pooled LR+ reaches 6.78 (95% CI, 4.87–9.44), which means that it is possible that ACL injury occurred in suspected cases when the MRI result was positive. Moreover, the pooled LR− had a value of 0.16 (95% CI, 0.13–0.20). In other words, there is a real possibility of excluding an ACL injury in suspected injured patients when the MRI result was negative. In addition, DOR represents a summary measure of the power of the test and the higher this measure, the better the performance of the inspection method^35^. The pooled DOR was 44.70 (95% CI, 32.34–61.79) in the present study, which predicts that the odds of obtaining a positive result using MRI are 44.7 times higher for an ACL injury than for an intact knee. In addition, the area under the curve (AUC) was 0.93, which indicates that MRI examination has a high diagnostic accuracy. Low, medium and appreciable accuracies of diagnosis are considered for AUC values ranging from 0.5 to 0.7, 0.7 to 0.9 and ≥0.9, respectively. The maximum AUC value of 1 predicts that the diagnostic test is perfect for differentiation in diagnostic test evaluation. In contrast, an AUC value < 50% indicates a poor performance of the diagnostic test^13^.

The MFI of MRI is one of the most important factors affecting accuracy of diagnosis. Smith *et al*. (2016) proved that there is no evidence that 3T scanners had superior diagnostic efficacy for ACL injury when compared with 1.5T machines^11^. Similarly, Phelan *et al*. (2015) and Smith *et al*. (2012) also reported that magnetic field strength had no significant effect on accuracy^12, 36^. Our results indicate that there are no significant differences in SE, SP, LR+, LR− and DOR between MFI greater than or equal to 1.5T and MFI below 1.5T (*p* = 0.85, *p* = 0.76, *p* = 0.84, *p* = 0.75, *p* = 0.84, respectively), which is not only consistent with the results of previous studies, but also corroborate previous studies.

Another important factor that affects the diagnostic accuracy is the MRI sequence. Oei *et al*. (2003) reported that improving the MRI sequence could improve diagnostic accuracy^37^. However, no study has yet compared the accuracy of diagnosis between different MRI sequences. Our meta-analysis provides evidence that there are no significant differences in SE, SP, LR+, LR− or DOR between CSs and CSs + PDWI (*p* = 0.95, *p* = 0.34, *p* = 0.53, *p* = 0.90, *p* = 0.45, respectively). Moreover, the SE of the two groups were equal (SE = 0.87). However, we found that the SP, LR+, LR− and DOR values were better in CSs + PDWI than in CSs.

In previous reviews, the impact of the study’s year of publication was found to be variable. Oei *et al*. (2003) reported that recent studies had better diagnostic accuracy than older studies^37^, which is likely due to improvements made in imaging technology such as the use of specific knee coils, improved sequences and radiologist familiarity with MRI over time. In contrast, Crawford *et al*. (2007) found that there is a negative trend in diagnostic accuracy with more recent studies^38^, which may be due to differences in the prevalence of ACL tears in the selected studies. They also reported that older studies had better methodological quality than recent studies. Therefore, they included all studies regardless of the year of publication. Our meta-analysis found that SP was significantly different between studies published during the periods 2006–2009 and 2012–2016 (SP = 0.93 vs. SP = 0.89, respectively; *p* = 0.04). Through a detailed reading of the literature included in the meta-analysis, we found that this was due to SP values of three articles that reached 100%^16, 19, 28^, which may be related to the MRI sequence or the specific knee coil used in their original study, such as fat-suppressed PD-weighted TSE-sequence and 3-minute three-dimensional isovoxel true FISP MR sequence or MRI devices using an eight-channel phased-array knee coil with coronal short tau inversion recovery sequence. On the other hand, there are seven studies that did not mention the sequence that was used and six of them were published during the 2012–2016 period. Meanwhile, our results indicated that there were no significant differences in SE (*p* = 0.08), LR+ (*p* = 0.09), LR− (*p* = 0.11) or DOR (*p* = 0.75) values between studies published during the 2006–2009 period and those during the 2012–2016 period. The SE and LR− values were better in recent studies than in older studies, while the LR+ and DOR values were better in older studies. Therefore, we could not draw any conclusion regarding which publication period produced better results. This aspect would need further investigation.

Our meta-analysis has not only updated, verified, supplemented and improved previous studies, but it has also provided an objective and systematic evaluation of the value of MRI diagnosis for ACL injury, including its diagnostic accuracy and methodology. Additionally, our research suggested new direction for future diagnosis experiments. Firstly, future studies should attempt when possible to use the standards for reporting of diagnostic accuracy (STARD) in their diagnostic tests, and try to evaluate in detail the authenticity, reliability and clinical importance of their diagnostic tests, in order to make their results more accurate, complete and conclusive^39^. Secondly, the diagnostic and control tests should be performed as soon as possible during the study process, and acquisition conditions clearly defined. Ultimately, the assessment of the test results should be double-blinded. Finally, by comparing different MFIs and the different sequences used for ACL injury, we provided reference and guidance for clinicians who choose MRI for patients with ACL damage.

Even though this meta-analysis showed optimistic results for the diagnostic accuracy of ACL injury, the outcomes should be viewed cautiously due to several limitations related to this meta-analysis. Firstly, the selected studies varied greatly in sample size, continuity of enrolled patients and patient race in addition to scanning conditions. Besides, the MFI parameter, the method used to blind participants and assessors or the familiarity of the radiologist were not mentioned in several studies that were included. Secondly, our method cannot identify an accurate cut-off point on the sROC curve, which is in agreement with other meta-analysis of diagnostic accuracy. The reason is that there is no precisely measured value for the MR image and a threshold is not used in clinical examination^13^.

In conclusion, current evidence of our meta-analysis indicates that MRI examination is able to provide appreciable diagnostic performance for DOR and AUC in the detection of ACL injury with high SE and SP (greater than 85%). Yet, there is not enough evidence to show that a higher MFI results in better diagnostic accuracy when MFI greater than or equal to 1.5T was compared with MFI below 1.5T. In addition, CSs + PDWI sequences are only slightly better than CSs, but without any statistical difference.

## Materials and Methods

### Inclusion and exclusion criteria

The inclusion and exclusion criteria were formulated based on the PICOS principles (participants, intervention, comparison, outcome and study design) of preferred reporting items for systematic reviews and meta-analyses (PRISMA)^40^. Studies relevant to the diagnosis of ACL injury by MRI and Arthroscopy were included. Inclusion criteria contained the following five conditions.

#### Participants and intervention measures

Patients suspected of having ACL injury/tear, examined by MRI and arthroscopy. Patients’ age, gender or race did not limit inclusion.

#### Comparison

MRI versus arthroscopy.

#### Outcomes

We obtained the pooled SE, SP, LR+, LR−, DOR and the sROC curve by extracting (directly or indirectly) the raw data (TP, FP, TN and FN results).

#### Study design

Prospective or retrospective study.

#### Languages and publication time

Studies in English published from January 1, 2006 to May 31, 2016 were included.

#### Exclusion criteria

Studies were excluded if they met one of the following conditions: (1) the type of article was a review, an abstract or a conference paper; (2) the study was performed on animals or cadavers; (3) the sample size of the study was less than 25 cases; (4) the raw data was not complete, thus preventing the calculation of TP, FP, FN or TN; (5) the patients were not examined using MRI and arthroscopy simultaneously; (6) clinical data were insufficient; (7) repeated reports came from the same team or the same set of data.

### Search strategy

Computer retrieval of English studies from PubMed, EMBASE, and Ovid databases, in addition to BIOSIS Previews and the Cochrane library was performed from January 1, 2006 to May 31, 2016. In addition, a manual retrieval was achieved based on references, magazines, ResearchGate, the national library reference service platform or by sending emails to authors. We used the following MeSH heading and keywords: magnetic resonance imaging AND anterior cruciate ligament AND arthroscopy.

### Screening and literature selection

The screening of the original literature should be strictly followed by the inclusion and exclusion criteria. There were four steps in the selection process. Firstly, the two researchers eliminated duplicated reports coming from the same team or the same set of data. Secondly, the two researchers selected the papers by reading titles and abstracts according to the inclusion and exclusion criteria. Thirdly, by evaluating the full text, the two researchers screened the potentially available studies conforming to the inclusion and exclusion criteria. Fourthly, to re-assess the full text, the two researchers chose the studies for which TP, FP, TN and FN could be extracted and calculated. The two researchers completed the screening process independently. When their opinions differed, they discussed the results until they reached the same conclusions.

### Data extraction

The two researchers designed a standardized abstract form, extracted data respectively and mutually checked their data. Disagreements relating to values or assessment were resolved by discussion. Extracted variables included: the author, the year of publication, the country where the study had been performed, the study designation, MFI, the number of samples, the demographic characteristics, the blinding process and TP, FP, TN, FN, SE and SP values.

### Quality evaluation

The methodological assessments of the quality of eligible studies were graded by two researchers independently, according to the QUADAS-2 tool (Agency for Healthcare Research and Quality, Cochrane Collaboration, and the U.K. National Institute for Health and Care Excellence)^41^, which is recommended for use in systematic reviews of diagnostic accuracy based on sources of bias and variation. The following four aspects are required to use the QUADAS-2 tool: (1) summarize the evaluation question; (2) develop the tool and produce evaluation with guidance; (3) construct a flow diagram for the original study; and (4) judge bias and applicability. The QUADAS-2 tool can provide obvious grades of bias and applicability of primary diagnostic accuracy studies. It comprises four significant domains including: (1) patient selection; (2) index test; (3) reference standard; and (4) the flow and timing. Each domain contains several signal questions used to help judge the risk of bias (low, high or unclear)^41^. The two researchers completed the screening process independently. Disagreement in the process of answering questions was discussed until consensus was reached. A final decision of “yes (satisfactorily elaborated)”, “no (unsatisfactorily elaborated)” or “unclear (data are insufficient making a judgment difficult)” was made by the researchers after systematic discussion. If the answers to all the signal problems were “yes”, a low risk of bias was attributed to the study; if the answers to all the signal problems had one or more “no” or “unclear” values, an unclear risk of bias was used; if the answers to all the signal problems contained at least one “no” but no “yes” answers, a high risk of bias was attributed. QUADAS-2 tabular and graphical display can be retrieved from the Web page, http://www.bris.ac.uk/quadas/quadas-2.

### Statistical analysis

Meta-Disc 1.4 for Windows (XI Cochrane Colloquium, Barcelona, Spain) statistical software was used for the heterogeneity test, outcomes combination and subgroup analysis^42^. Stata 14.0 (Stata Corp., College Station, TX, USA) was used for publication bias. A two-sided statistical test was considered suitable and statistical significance was set at *p* < 0.05.

Heterogeneity is usually caused by threshold and non-threshold effects. If the threshold effect exists, the pairs of accuracy estimates (SE and SP, or LR+ and LR−) are negatively correlated (or SE is positively correlated with 1 - SP), or vice versa; the accuracy estimates distribution of each independent study shows a typical “shoulder arm” shape in the sROC curve; or the Spearman correlation coefficient reflects a significant relationship between the logit of sensitivity and the logit of 1-specificity according to *p* and *r* values. Besides the threshold effect, non-threshold effects also cause heterogeneity, including population (such as disease severity and complications), test conditions (such as different technologies, laboratory tests and operators), standard tests and so on. This can be detected through Chi-square and Cochran-Q statistical tests. If non-threshold effects exist, then *p* < 0.05^43^.

A fixed effects model was used with no heterogeneity among individual studies when *p* > 0.05 and *I* ^2^ < 50%. This calculation model of the combined effect indicated that all the variation in the eligible studies was caused by chance. In other words, the model assumed that the measurements over all effects were from the same population. Otherwise, a meta-regression analysis can be used to explore the potential factors of heterogeneity (such as the participants, the test, the standard test, the methodological characteristics, *etc*.). When persistent heterogeneity among eligible studies exists, a random effects model can be used to analyze the sampling error (variance) and the variance of the research with *p* < 0.05 and *I* ^2^ ≥ 50%^44^, and estimate the uncertainty of the results by 95% CI, because of the clinical importance of some indices. This calculation model could give a wider CI than the fixed effects model when the heterogeneity is caused by other potential factors.

A fixed effects model with the Mantel-Haenszel method or a random effects model with the DerSimonian-Laird method was applied to calculate the pooled SE, SP, LR+, LR− and DOR with 95% CI based on the level of heterogeneity of the eligible study presenting in forest plots. The sROC curve with 95% CI was established by combining data, which could evaluate the potential association between SE and SP in a metamorphic approach. A value of ½ was added to all cells of studies when data with a zero value appeared.

A subgroup analysis was subsequently assessed in a more homogeneous group according to MFI (≥1.5T versus <1.5T), year of publication (2006 to 2009 versus 2012 to 2016) and MRI sequences (CSs versus CSs + PDWI), which was comprised more than 3 studies. Differences between subgroups were calculated through *t* test or rank sum test^45^.

A Deeks’ funnel plot asymmetry test was used with a significance level set at *p* < 0.05 to predict the existence of publication bias^46^, which is of great concern for meta-analysis of diagnostic studies.

### Data Availability

The datasets generated during and/or analyzed during the current study are available from the corresponding author on reasonable request.
